# Effects of Acute Probenecid Administration on Histopathological and Functional Outcomes after Spinal Cord Injury in Rats

**DOI:** 10.1089/neur.2025.0044

**Published:** 2025-05-26

**Authors:** Toru Asari, Sunao Tanaka, Damien D. Pearse, Juan Pablo de Rivero Vaccari, Toshitada Sawada, Yasuyuki Ishibashi, Robert W. Keane, W. Dalton Dietrich

**Affiliations:** ^1^ Department of Neurological Surgery, The Miami Project to Cure Paralysis, University of Miami Miller School of Medicine, Miami, Florida, USA.; ^2^ Department of Orthopaedic Surgery, Hirosaki University Graduate School of Medicine, Hirosaki, Japan.; ^3^ Department of Cellular Physiology and Molecular Biophysics, University of Miami Miller School of Medicine, Miami, Florida, USA.; ^4^ Department of Orthopaedic Surgery, Takaoka Seishikai Hospital, Takaoka, Japan.

**Keywords:** inflammasome, probenecid, Pannexin-1, spinal cord injury

## Abstract

Spinal cord injury (SCI) triggers an inflammatory response that is partially mediated by the inflammasome and the production of pro-inflammatory cytokines. We have previously shown that pannexin-1 is involved in the activation of the inflammasome, and that probenecid inhibits this caspase-1-mediated inflammatory process. In this study, we employed an *in vivo* model of contusive SCI to investigate the therapeutic effect of acute probenecid administration on histopathological and functional outcomes following SCI. Adult female Fischer rats (*n* = 46) underwent moderate thoracic SCI produced by dropping a 10 g weight from a height of 12.5 mm onto the exposed cord at T9, assigned three different groups, PBS administration group, and 1, 10, 100 mg/kg probenecid group, those were injected subcutaneously 15 min and 12 h after SCI. The sham group (*n* = 11) was the group that only had a laminectomy and did not have SCI. Histopathological analysis by Luxol Fast Blue/hematoxylin and eosin staining revealed that the penumbra volume was significantly reduced in the probenecid 100 mg/kg group compared with the PBS group. CatWalk gait analysis was performed at 7 weeks after SCI, which showed significant differences in coordination between the PBS and the probenecid 100 mg/kg-treated groups. Acute administration of probenecid after SCI resulted in the preservation of penumbra formation and coordination function in a thoracic SCI rat model. This report suggests that probenecid, an inhibitor of pannexin-1, has the potential to prevent secondary injury after SCI and improve outcomes following SCI.

## Introduction

The incidence of spinal cord injury (SCI) is 26.5 cases per 1 million inhabitants.^1^ Currently, nonpharmacological, pharmacological, cell-based, and mixed therapies are being investigated in clinical trials.^2^ However, no proven, strongly effective single therapeutic method exists that has demonstrated a positive effect on neurological outcomes after SCI, thus emphasizing the need for continuous research in the pathophysiology and treatment of this serious problem.^3^

Central nervous system (CNS) responses to acute focal damage can be divided into three distinct phases: (1) cell death and inflammation, (2) cell proliferation, and (3) tissue remodeling.^4^ Regarding the inflammatory response, pro-inflammatory cytokines such as interleukin (IL)-1β and IL-18 have been reported to play a deleterious role in the pathophysiology of SCI.^5^ Following SCI, the inflammasome has been shown to activate IL-1β and play a role in humans and rodents after injury.^6,7^ The inflammasome is a multiprotein complex involved in the activation of caspase-1. Once activated, caspase-1 processes the pro-inflammatory cytokines IL-1β and IL-18.^8^ It has been previously shown that the NOD-like receptor protein-1 (NLRP1), inflammasome forms protein–protein interactions with Pannexin-1 (Panx1) and the purinergic receptor P2X7 in rat neuronal cells in culture.^9^ In addition, it has been shown that P2X4 plays a critical role in mediating innate neuroinflammatory events after SCI.^6^

Probenecid is a powerful nonspecific inhibitor of the Panx1 channel^10^ and the inflammasome.^9^ For decades, probenecid has been used as a commercial drug in the treatment of gout, and it is thought to act on an organic anion transporter, thereby affecting urate excretion in the kidney by blocking urate reuptake.^11,12^ Recently, the effects of probenecid on traumatic SCI have been investigated.^13–15^ In a study by Qi and colleagues,^15^ they demonstrated that probenecid administered after T9/10 cord injury of Sprague Dawley (SD) rats reduced inflammasome activation through detailed immunoblotting, immunohistological, and flow cytometric studies in 3 days after injury. Also, improvements of histopathological and behavioral outcomes have been reported 6 weeks after injury.^15^ Given the deleterious effects of inflammasome activation following CNS injury,^16^ and the role that Panx1 plays in the inflammasome,^9^ inhibition of Panx1 by probenecid is expected to prevent secondary damage after SCI.

In a previous SCI study, Qi et al. administered probenecid within 3 h after injury, followed by daily intraperitoneal injections (1 mg/kg). Silverman et al.^10^ reported that probenecid completely inhibited currents in the Pannexin 1 channel at 1 mM (0.001 mol/L). But previously, different doses and different routes of administration of probenecid have been tested in other experiments conducted on rats.^17,18^ Thus, the optimal dosage and timing of administering probenecid to prevent secondary spinal cord damage and improve functional outcomes has not been clarified.

In this study, we hypothesized that probenecid administered immediately after SCI might inhibit the acute activation of the inflammasome, thereby preserving glial scarring of the spinal cord. We investigated the administration of probenecid in different doses on the day of injury and performed a multifaceted functional evaluation in the rat SCI model up to 7 weeks after injury. Our current findings provide additional support for the use of probenecid in reducing inflammasome activation after moderate SCI, as well as in attenuating histopathological damage and improving motor function.

## Materials and Methods

### Spinal cord injury in rats

Two adult female Fischer rats (180–200 g; *n* = 57) were housed per cage, where they had access to food and water *ad libitum*. All study protocols were approved by the University of Miami Institutional Animal Care and Use Committee, in accordance with the NIH and the Guide for the Care and Use of Laboratory Animals. For injury procedures, rats were anesthetized with 2% isoflurane and a mixture of 30% oxygen. Verification of levels of anesthesia was assessed by the corneal reflex and withdrawal reflex, which were used to stimulate the hindlimbs. The rat’s back was shaved and scrubbed with chlorhexidine scrub solution. A 2 cm longitudinal skin incision was centered over the T9 spinous process along the midline. Muscles and ligaments were dissected and retracted laterally. T8–10 vertebrae were exposed, and a dorsal laminectomy was performed at T9 to expose the dura mater.

A moderate contusion injury was induced utilizing the MASCIS weight drop device developed at New York University.^19^ Moderate injury was produced by dropping a 10 g weight from a height of 12.5 mm onto the exposed cord.^19^ Animals were excluded when variables exceeded 0–6% for height and velocity error and 1.25–1.75 for the compression distance value. The sham group (*n* = 11) received only a laminectomy at T9, and did not injure the spinal cord. After the injury, muscles were sutured in layers, and the skin was closed using Michel clips. The rats were returned to their cages with temperature regulated for the following 24 h and allowed access to water and food along with a heating pad. Animals were injected with gentamicin (5 mg/kg) for 3 days and 6 mL of Ringer’s solution subcutaneously twice daily for 3 days. The analgesic, buprenorphine (0.03 mg/kg), was administered subcutaneously for 2 days after surgery. Bladders were expressed twice daily for 3–10 days until they began self-excretion.

### Drug administration

Rats were randomly assigned to be treated with different concentrations of probenecid (1 mg/kg, *n* = 12; 10 mg/kg, *n* = 10; 100 mg/kg, *n* = 12). Probenecid was administered subcutaneously at a total volume of 1 mL, adjusted with PBS to the respective dose according to body weight. Controls were treated with an equal amount of PBS (*n* = 12). The injections were performed 15 min and 12 h after SCI in a double-blinded manner.

### Basso, Beattie, and Bresnahan and the BBB subscoring system

Two nonbiased observers recorded hindlimb locomotor function using the Basso, Beattie, and Bresnahan (BBB) scoring system and the BBB subscoring system.^20,21^ Individual rats were placed in a metal elliptical enclosure and allowed to acclimate to their surroundings. BBB scoring tests were conducted at baseline (before surgery), 24 h after surgery, and then once a week until 7 weeks after surgery. BBB subscoring tests were conducted at baseline and from the second postoperative week, with assessments conducted weekly until 7 weeks after surgery.

### CatWalk gait analysis

The walking coordination was analyzed during overground locomotion using the CatWalk device (Noldus Information Technology Inc., Leesburg, VA), during which the walking patterns of all four limbs were filmed from underneath while the rats crossed an enclosed walkway.^22^ Catwalk gait analysis evaluates both static and dynamic locomotor parameters, such as stride length, base of support, interlimb coordination, and swing/stance phases. We evaluated the mean coupling between the right front limbs and left hindlimbs, referred to as “right forelimb (RF)->left hindlimb (LH) mean,” and the mean of the linkage of the movements of the limbs on the diagonal. Coupling was calculated with the following formula: Coupling = (initial contact time of target limb − initial contact time of anchor limb)/(step cycle of anchor limb) ×100. The lower the value, the closer in time the two limbs make contact with the walkway.^23^ Catwalk gait analysis was also performed before surgery and at the study’s endpoint (7 weeks).

### Grid walking test analysis

Grid walking test was used to evaluate sensory-motor coordination of the forelimb and hindlimbs. For the grid walking test, deficits in descending fine motor control were examined by assessing the rats’ ability to navigate across a 1 m-long runway enclosed in a clear Plexiglass chamber with irregularly spaced gaps (0.5–5.0 cm) between round metal bars.^24^ Data were assessed by evaluating the percentage of footfall hindlimb errors per crossing. Grid walking test was also performed before surgery and at the endpoint of this study (7 weeks).

### Histopathological assessment

At 7 weeks after SCI, the rats were anesthetized with 3% isoflurane, 70% N_2_, and 30% O_2_, and then injected with ketamine (100 mg/kg) and xylazine (10 mg/kg) intraperitoneally. They were then transcardially perfused and processed for Luxol Fast Blue and counterstained with hematoxylin and eosin for lesion volume analysis as described.^7^

### Quantitative analyses of stained tissue sections

**FIG. 1.**
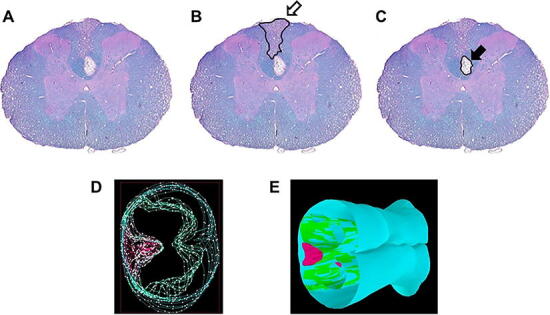
Histopathological analysis. **(A)** Representative coronal section of the injured spinal cord after LFB staining, counterstained with H&E. (**B)** The white arrow indicates the lesion of penumbra. (**C)** The black arrow indicates the cavity lesion. **(D)** The image after merging the traced white matter, gray matter, penumbra, and cavity every 600 μm throughout the total 30 mm segment. (**E)** The merged sections were automatically constructed into the 3-dimensional image, and the volume of each lesion was estimated with Neurolucida software. H&E, hematoxylin and eosin; LFB, Luxol Fast Blue.

To investigate the effects of the probenecid on myelin preservation and suppression of necrosis after SCI, stained tissue section was analyzed every 600 μm throughout the total 30 mm segment under the microscope (BX51, Olympus America Inc., NY). The lesions of white matter, gray matter, penumbra, and cavity areas were traced in 13 slices per specimen using Neurolucida 11.03 software (MBF Bioscience, Williston, VT) (Fig. 1A–C). The area and total volume of each section were automatically estimated by the Neurolucida Explorer software version 4.50.4 (MBF Bioscience, Williston, VT) (Fig. 1D, E), as previously described.^25,26^

### Statistical analysis

Statistical analysis of data among groups was performed using two-way ANOVA for BBB score and the BBB subscore, and the grid walking test. A one-way ANOVA followed by Dunnett’s post hoc test was used for the CatWalk test, as well as for histological area and total volume analysis. General linear mixed analysis with pairwise comparisons was performed between the sham group and the PBS group in the CatWalk gait analysis. The Wilcoxon signed-rank test was used to compare the change in percentage of foot falls between the baseline (0 weeks) and 7 weeks after SCI (7 weeks) among each group. All statistical analyses were performed using the SPSS software version 12.0 (SPSS Inc., Chicago, IL). A *p* value of <0.05 was considered statistically significant.

## Results

One animal in the 1 mg/kg probenecid group was sacrificed because of unknown sickness after SCI, and excluded from both behavioral and histological evaluation. Due to the poor quality of the staining specimens, two specimens from the PBS group were excluded from the histological analysis.

### Acute probenecid administration did not improve BBB and BBB subscore

**FIG. 2.**
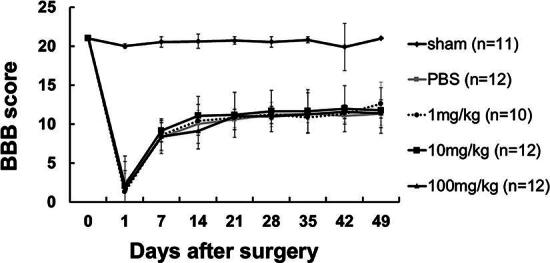
Basso, Beattie, and Bresnahan (BBB) score. The mean ± standard deviation (S.D.) values of the BBB score up to 7 weeks in all five groups. Sham group means laminectomy only, and there was no spinal cord injury. The numbers indicate the probenecid dosage concentrations. There was no significant difference in the BBB score between groups except for the sham group at any individual time point.

One day after SCI, severe motor deficits were observed in all groups by the BBB scoring scale. At 2 and 3 weeks after SCI, locomotor function recovered gradually, followed by a plateau by week 7. The mean ± standard deviation values of the BBB score at 7 weeks in all four groups (PBS, 1 mg/kg, and 10 mg/kg, 100 mg/kg) were 11.3 ± 1.7, 12.6 ± 2.7, 11.7 ± 2.9, and 11.4 ± 0.6, respectively (Fig. 2). There was no significant difference in the BBB score between groups at any individual time point.

**FIG. 3.**
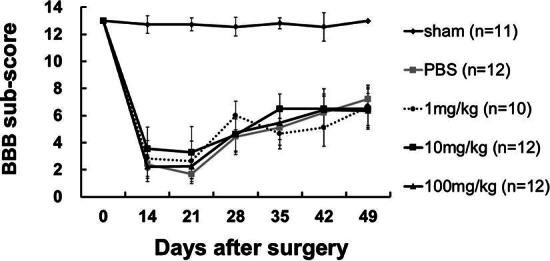
Basso, Beattie, and Bresnahan (BBB) subscore. The mean ± standard deviation (S.D.) values of the BBB subscore up to 7 weeks in all five groups. Sham group means laminectomy only, and there was no spinal cord injury. The numbers indicate the probenecid dosage concentrations. There was no significant difference in the BBB score between groups except for the sham group at any individual time point.

The mean values of the BBB subscore at 7 weeks in all groups (PBS, 1 mg/kg, 10 mg/kg, and 100 mg/kg) were 7.2 ± 1.0, 6.7 ± 1.4, 6.5 ± 1.4, and 6.4 ± 1.2, respectively (Fig. 3). There was no significant difference in the BBB score between groups at any individual time point.

### Acute probenecid administration improved coupling motion and sensory-motor coordination after SCI in rats

**FIG. 4.**
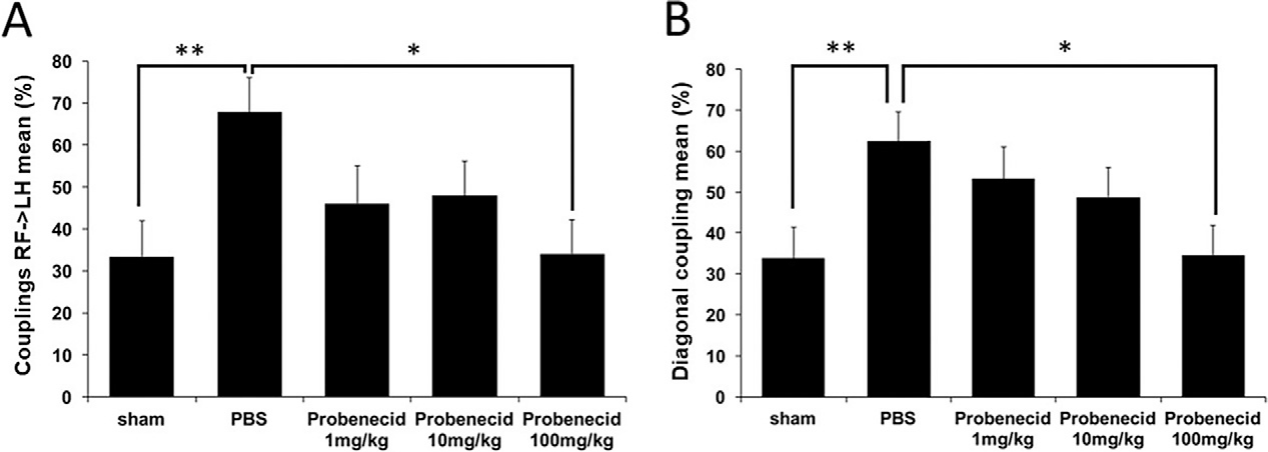
CatWalk gait analysis. CatWalk gait analysis was performed at 7 weeks after spinal cord injury (SCI). **(A)** Gait analysis of coupling ratios of right forelimb (RF) to left hindlimb (LH) percentage. **(B)** Couplings diagonal percentage. Data presented as mean ± S.E.M. In the sham group, rats underwent only a laminectomy. There are significant differences between the sham and PBS groups, as well as a notable difference between the PBS and the probenecid 100 mg/kg-treated groups. Sham: *n =* 11, PBS: *n =* 12, 1 mg/kg *n =* 9, 10 mg/kg: *n =* 12, 100 mg/kg: *n =* 12. *p < 0.05, **p < 0.01.

**FIG. 5.**
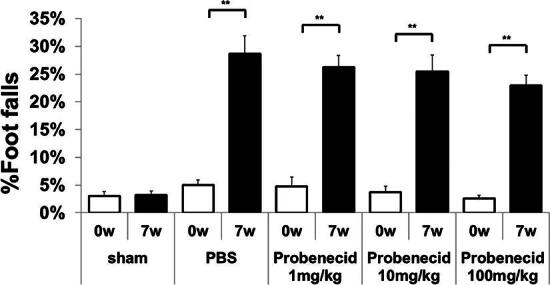
Grid walking test analysis. The percentage of foot falls in baseline (0 w) and 7 weeks after injury (7 w). Data are presented as mean ± S.E.M. There was significant difference among baseline (0 w) and 7 weeks after injury (7 w) in PBS, 1 mg/kg, 10 mg/kg, 100 mg/kg probenecid groups, no significant difference was seen between groups at 7 weeks after SCI except for sham. Sham: *n =* 11, PBS: *n =* 12, 1 mg/kg *n =* 9, 10 mg/kg: *n =* 12, 100 mg/kg: *n =* 12. **p < 0.01.

Catwalk gait analysis was assessed at baseline and 7 weeks after SCI. We compared the couplings of RF->LH mean percentage and couplings diagonal mean percentage in probenecid-treated SCI rats compared with the sham and PBS groups at 7 weeks after SCI (Fig. 4A, B). There was a significant difference between the sham and PBS groups, and between the PBS and probenecid 100 mg/kg group. The Gridwalk test revealed a trend for probenecid-treated rats to exhibit a smaller percentage of footfalls as probenecid concentrations decreased at 7 weeks after SCI, although there was no significant difference between groups (Fig. 5).

### The lesion volume of penumbra was significantly smaller in the group of 100 mg/kg probenecid administration after SCI

**FIG. 6.**
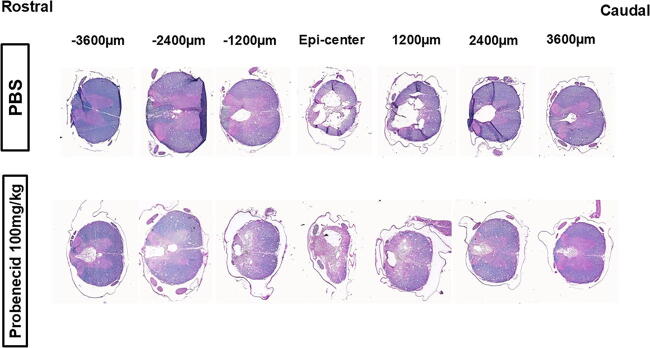
Representative coronal section series of the injured spinal cord. A typical example of the PBS or probenecid (100 mg/kg) groups is shown. A stained tissue section was analyzed every 600 μm throughout the total 30 mm segment, centered on the most injured section (Epi-center), under the microscope. This figure presents section for every 1200 μm. Luxol Fast Blue (LFB) staining, counterstained with hematoxylin and eosin (H&E).

**FIG. 7.**
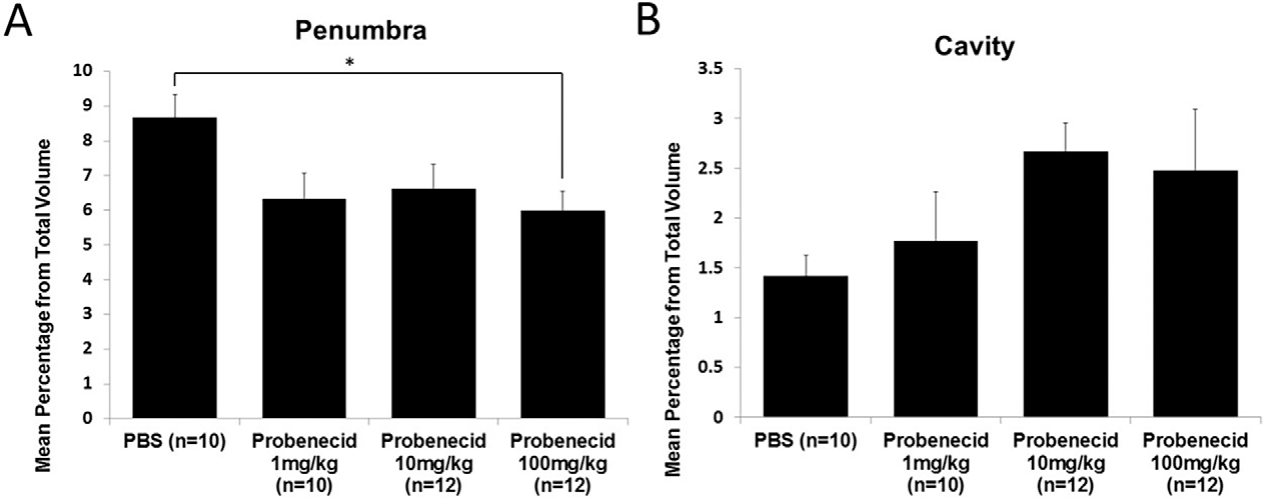
Quantitative histopathological assessment of the penumbra and cavity volume. **(A)** The penumbra volume was significantly smaller in the probenecid 100 mg/kg group compared with the PBS group. **(B)** There was no significant difference in the cavity volume among those four groups. Data presented as mean ± S.E.M. PBS: *n =* 10, 1 mg/kg *n =* 9, 10 mg/kg: *n =* 12, 100 mg/kg: *n =* 12. *p < 0.05.

Representative coronal section series are shown in Figure 6. Quantitative data for the total 30 mm cord segment, centered on the epicenter of the injured spinal cord, are presented in Figure 7. The lesion volume ratio at the penumbra was significantly smaller in the group that administered 100 mg/kg probenecid compared with the PBS-treated group (Fig. 7A). There was no significant difference in the cavity volume ratio between the PBS and each of the probenecid-treated animals (Fig. 7B).

## Discussion

In this study, we demonstrated that subcutaneous probenecid injection at 100 mg/kg, 15 min and 12 h after SCI, reduced the lesion volume of the penumbra and prevented the worsening of interlimb gate coordination in a moderate thoracic SCI model in rats. Although locomotor function, evaluated by BBB and BBB subscore, did not improve with the administration of probenecid, this result suggests that simple acute-phase administration of probenecid suppresses the inflammatory response at the site of SCI and prevents secondary damage to the spinal cord after injury.

The Panx1 channel was discovered in 2000, and it is an adenosine triphosphate (ATP) release channel involved in the propagation of intercellular calcium waves.^27–30^ After SCI, ATP released from dying cells activates the P2X4 receptor. P2X4 then causes an efflux of potassium, resulting in increased extracellular potassium concentration. High extracellular potassium opens the Panx1 channel, leading to additional ATP release. High extracellular levels of ATP activate the P2X7 receptor and the inflammasome, leading to the processing of pro-IL-1β and pro-IL-18.^5,9,16^ Panx1 is a major ATP release channel, expressed in most cells, releasing ATP via a nonexocytotic pathway.^29^ Panx1 interacts with the inflammasome and with P2X7 receptors. In the process of inflammasome activation, Panx1-mediated ATP release is at an early stage in which Panx1 acts as a signal amplifier. Therefore, Pan1 is an important target for the development of therapeutic methods. The Panx1 inhibitor probenecid is already FDA approved to treat gout with few side effects. In the process of inflammasome activation, high extracellular potassium opens Panx1, leading to caspase-1 activation *in vitro*.^9^ Moreover, caspase-1 activation induced by high extracellular potassium is inhibited by blocking Panx1 with probenecid in neurons and astrocytes.^9,31^

Silverman et al.^10^ reported that probenecid completely inhibited currents in the Pannexin 1 channel at 1 mM (0.001 mol/L), with an IC 50 of ∼150 μM, suggesting that concentrations in the range of 150 μM to 1 mM were considered suitable for the effect of probenecid. Therefore, a subcutaneous dose of 1 mg/kg was considered sufficient to inhibit Pannexin1 channels immediately after SCI. However, since different doses of Probenecid have been administered in previous studies,^17,18^ we aimed to explore multiple probenecid dosages on the effects on functional and structural outcomes after SCI. Therefore, we administered 10 and 100 mg/kg to investigate the effect of these doses on the prevention of secondary SCI. Furthermore, to determine the extent to which inhibition of pannexin1 channels immediately after SCI would reduce the spread of secondary injury, only two doses were administered: one immediately after injury (15 min) and another 12 h later.

Our study suggests that a 100 mg/kg subcutaneous probenecid injection in SCI rats is an effective approach for preventing secondary injury. The results of this study also suggest the importance of administering probenecid as early as possible after injury to inhibit inflammasome activation that occurs immediately after SCI. Xiong et al.^32^ administered 1 mg/kg probenecid intraperitoneally to a mouse model of cerebral ischemic stroke and demonstrated a decrease in the lesion size of the penumbra. They compared two different protocols for administration of probenecid, pre- and postischemia treatment, and only post-ischemic treatment alone. Interestingly, this study reported that pre- and postischemia treatment of probenecid significantly improved neurological deficits. In contrast, postischemic treatment did not show significant outcome differences compared with vehicle-treated animals. The activation of inflammasome occurs early after injury, thus suggesting that a pretreatment protocol with probenecid might be more beneficial in reducing inflammasome activation. On the other hand, Qi et al.^15^ had reported administering probenecid within 3 h after SCI, followed by daily intraperitoneal injections (1 mg/kg) until 7 weeks after injury, significantly improvement of BBB score was seen from 2 to 7 weeks after injury compared with the PBS-injected group. Those findings indicate that inflammasome activation persists not only early after SCI but also for weeks afterward, and that continued suppression of inflammasome activation during this period is necessary to most efficiently improve motor function.

In this study, the administration of probenecid after SCI reduced the lesion volume of the penumbra but did not reduce the volume of the cavity. The penumbra is the tissue surrounding the injured area and has been reported as a potentially treatable and reversible lesion of tissue, characterized by reduced cerebral blood flow, located adjacent to the focal ischemic or injured area. In this region, blood flow and oxygen supply are reduced, and cells are under stress, and it has the potential to recover with timely intervention in the hypoperfusion.^33^ There had been reports of increasing activated glial and inflammatory cells in the penumbra of the SCI lesion.^34^ Probenecid is an inhibitor of the Panx1 channel, and it is expected to suppress a series of inflammasomes, thereby inhibiting the formation of gliosis scars after SCI. Since probenecid itself does not promote nerve regeneration, it is assumed that the volume of the cavity where nerve necrosis occurred did not change. We propose that suppressing the activation of the inflammasome, which persists for several weeks after injury, there is improved wound healing that may minimize the inhibition of nerve recovery, leading to improved motor function, as reported by Qi et al.^15^

As shown by the results of tissue staining, a penumbra was present on the dorsal side of the spinal cord, which had been damaged from the dorsal side. Anatomically, there is the descending corticospinal tract, which selectively and complexly modulates sensory information in the dorsal horn of the rat spinal cord.^35^ These findings are consistent with the notion that the volume reduction of the dorsal penumbra of the spinal cord prevented secondary damage to the normal neural tissue of the dorsal spinal cord, thereby improving the coordination of the forelimb and hindlimb, as assessed by the CatWalk test in this study and tendency to reduce percentage of foot falls in grid walk test.

There are other reports about the effectiveness of probenecid to decrease inflammasome activation for various diseases. Mawhinney et al. reported that treatment with probenecid reduced NLRP1 inflammasome activation in the hippocampus and improved spatial learning performance in aged rats.^36^ In addition, the importance of Panx1 in neuronal cell death comes from a study on an animal model of Crohn’s disease. In this study, probenecid protected enteric neurons and preserved functional control of the colonic musculature *in vivo*.^37^ Therefore, probenecid may be potentially useful as a therapeutic drug for various diseases associated with inflammasome activation.

NLRP1, NLRP2, NLRP3, and absent in melanoma 2 (AIM2) inflammasomes have been well characterized after CNS injury. The NLRP1 inflammasome is present in motor neurons of the spinal cord and in brain cortical neurons,^7,38,39^ and the NLRP2 inflammasome is present in astrocytes.^31^ In addition, the NLRP3 inflammasome is present in microglia/macrophages,^40^ and the AIM2 inflammasome in neurons.^41^ Previously, our group analyzed the role of P2X4 receptors on inflammasome activation after SCI using P2X4 knock-out (KO) mice.^6^ P2X4 KO mice showed a significant decrease in inflammasome activation, pro-inflammatory cytokine production, inflammatory cell infiltration, and improved locomotor function as well as histopathological outcomes after SCI. These findings indicated that blocking neuronal P2X4 after SCI could be a therapeutic method to reduce the activation of inflammasomes and loss of neurological function after CNS injury. However, P2X4 selective antagonists have not been tested in the context of SCI yet.^42^ Probenecid has already been used as a commercial drug in the treatment of gout, making it the closest to a practical application of the drug as an inhibitor of the inflammasome in the treatment of SCI.

In summary, the current study reports that the administration of probenecid twice, once immediately after injury (15 min) and again 12 h later, reduced the lesion of the penumbra and prevented the deterioration of coordination in the thoracic moderate SCI rat model. These findings support previous research in the SCI field showing that probenecid treatment may be protective by reducing inflammasome signaling, thereby protecting from secondary injury of the spinal cord. Future research is needed to evaluate this therapy in other injury models to confirm the beneficial effects of probenecid in SCI.

### Transparency, rigor, and reproducibility

This article is designated as a translational therapeutic study as it involves nonhuman animal subjects with characteristics relevant to human SCI. This study was not formally registered; however, the proposal describing the work was reviewed extensively by multiple committees and subsequently updated. The proposal received funding from the Miami Project to Cure Paralysis, and the knowledge was in the public domain. The analysis plan was not formally preregistered, Drs. Asari and Tanaka, as team members with primary responsibility for the analysis, certify that the analysis plan was prespecified prior to initiation of the study. A power analysis based on pilot data and previous publications was used to set the desired effect size at 0.7. A sample size of *N =* 10–12 for the behavior and histology outcomes was calculated using G*Power3.1 (power set at 0.80 and alpha at 0.05). The investigators were blinded to the experimental groups for all data analysis. The probenecid dose was based on previous work in the laboratory. All materials required to perform the study are available from commercial sources. The experimental injury model is an established standard in the field. The sample sizes reflect the number of independent measurements and are comparable to those in previous reports using the same model. Correction for multiple comparisons was performed using GraphPad Prism. Data and analytic code from this study are available from the corresponding author. Materials used to conduct the study are not publicly available.

## Abbreviations Used

BBB: Basso, Beattie, and Bresnahan
SCI: Spinal cord injury
SD: Sprague Dawley
